# Digital Storytelling as an Intervention for Older Adults: A Scoping Review

**DOI:** 10.3390/ijerph20021344

**Published:** 2023-01-11

**Authors:** HeeKyung Chang, YoungJoo Do, JinYeong Ahn

**Affiliations:** 1 College of Nursing, Gerontologic Health Research Center in Institute of Health Science, Gyeongsang National University, Jinju-si 52727, Gyeongsangnam-do, Republic of Korea; 2 College of Nursing, Gyeongsang National University, Jinju-si 52727, Gyeongsangnam-do, Republic of Korea; 3 Gerontologic Health Research Center in Institute of Health Science, Gyeongsang National University, Jinju-si 52727, Gyeongsangnam-do, Republic of Korea

**Keywords:** digital, storytelling, intergeneration, older adults, review

## Abstract

The population of older adults is rapidly increasing worldwide. Owing to fewer interactions between generations, older adults experience ageism and various psychological issues, such as depression and loneliness. Digital storytelling (DST) has the potential to share vivid lived experiences, support the forming of social relationships, and lead to improved well-being. This scoping review examines the potential psychosocial benefits of individual DST interventions for older adults and people with dementia. We adopted the methodological framework for scoping reviews outlined in the Joanna Briggs Institute’s (JBI) manual. A scoping review was performed using the following bibliographic databases: Web of Science, PubMed, Cochrane Library, CINAHL, Research Information Sharing Service, and National Assembly Library. There were 395 references retrieved, of which 19 articles were selected after applying inclusion and exclusion criteria. Our findings revealed that the most common effects of DST on older adults included the promotion of mental health, an increased amount of meaningful community connections, greater digital literacy, the mitigation of negative ageism, and enhanced intellectual ability. We suggest randomized controlled trials are conducted to confirm the efficacy of intergenerational DST intervention and the effects of DST interventions at multilevel outcomes, including the community level.

## 1. Introduction

Storytelling is a natural and universal form of communicating [1]. Historically, knowledge and skills have been bequeathed via word of mouth. As stories can be modified and added during delivery, the speaker’s and listener’s imagination is infused for future re-telling, thereby creating potentially different stories [2]. Digital storytelling (DST) is a creative way for people to share their stories that involves fusing digital media with voices, images, video, and music [3]. This allows for interactions across diverse populations, broadening the conversation window and increasing social connectivity for older adults whose scope of activities is gradually narrowing [4].

Older adult populations are rapidly increasing. In 2050, one out of four persons will be 60 years or older [5]. The expanding older population has generated a discourse about healthy aging. Healthy aging incorporates an individual’s physical and mental abilities in order to promote well-being in old age, and simultaneously seeks to develop and maintain their physical, social, and policy environments [6]. It requires a holistic and integrated approach that encourages creative expression, participation in social activities, lifelong learning, maintenance of individual abilities, disease prevention, and physical health promotion [7]. To ensure the healthy aging of older adults [7], it has been suggested that their quality of life should be improved; social networks and positive social interactions are such factors that can enhance quality of life. Thus, solving the social issues of older adults, such as social isolation and loneliness, is necessary for healthy aging [7,8]. Older adults can benefit from a positive digital world approach when tackling this societal issue [6]. For example, older adults’ use of information communication technology (ICT) devices positively impacted mental health and subjective well-being by reducing loneliness and increasing autonomy [9]. Positive utilization of ICT is reported to boost social engagement interaction and foster a sense of connection through contact with older people and generations [6,8,10]; thus, it can be effective in resolving societal issues such as social isolation and loneliness. Older adults’ access to digital technology expands their digital world and enhances their ability to utilize these devices and gain more digital literacy, which can improve their mental health and quality of life [8]. One concept for supporting healthy aging in older adults is DST activity [10].

In DST, older adults build a tale using their own language and expressions to express themselves utilizing DST [2,3,11]. Using multimedia technology, DST includes the fusing of pictures, audio, and narration to produce a film that represents one’s lived experiences. Older adults can become digital creators and develop their imagination and speech skills by sharing their stories with others [4]. By narrating stories to the younger generation, participating in DST, and expressing their identities and personalities, older adults with early-stage dementia were shown to have improved self-efficacy and depression symptoms [11]. Moreover, when older adults participated in DST, they maintained their memory, positively affecting their overall life. The digital literacy of older adults could be improved by using ICT to increase the quality of social interactions [3,11]. In a previous study, older adults who used tablet PCs could better operate electronic devices, had higher levels of self-efficacy and confidence, and had more energy due to their active participation in numerous tasks [12]. The use of broadly defined DST to enhance older adults’ health is a growing field of research.

Review studies confirmed the effects of personal reminiscence therapy [13] and autobiography [14] by limiting the participants to older adults with dementia; however, neither of the two studies [13,14] included digital technology. Rincon [15] performed a systematic review of DST studies but retrieving individual papers or verifying their subjects was challenging because the articles were integrated for each study purpose. Stargatt et al. [16] also conducted a systematic review of DST, but the results were limited as only autobiographical experiences and health-related outcomes were assessed. To know more about the DST intervention process and better understand its effects on older adults, it is necessary to first identify the concepts and structures needed in DST interventions and to generate a potential list of attributes before designing interventions for older adults. We confirmed that a scoping review is the best approach to answer these research questions as the knowledge needs that prompted the project are congruent with the kinds of outputs a scoping review produces [17,18]. This scoping review could guide the development of more effective DST interventions for older adults. Our primary objective for this review is to frame the structures that can be used for applications of DST and to identify the effects of DST intervention through evidence synthesis.

## 2. Materials and Methods

We conducted a scoping review following Joanna Briggs Institute (JBI)’s Manual for Scoping Reviews [19]. It comprises the following consecutive stages: identifying the research question; identifying relevant studies; study selection; charting the data; and collating, summarizing, and reporting results. The Preferred Reporting Items for Systematic Reviews and Meta-Analyses extension for scoping reviews (PRISMA-ScR) checklist guided this review [20]. The protocol for this scoping review has not been registered or published.

### 2.1. Research Questions

This review answers the following questions:

1. How is a DST intervention structured and applied to older adults?
2. What are the effects of DST intervention on older adults?

### 2.2. Identifying Relevant Studies

We used the JBI reviewer’s manual and PRISMA-ScR guidelines to develop a comprehensive search strategy [19,20]. A broad systematic search was performed in December 2021 using the following databases: the Web of Science, PubMed, Cochrane Library, the Cumulative Index to Nursing and Allied Health (CINAHL), the Research Information Sharing Service, and the National Assembly Library.

The search terms were chosen to describe the population characteristics and the activities necessary for the review. The following syntax describes the terms that were used to search for the studies: (story*) AND (digital* OR ICT OR “artificial intelligence” OR online OR media OR mobile OR platform) AND (elder* OR older* OR dementia OR Alzheimer OR “cognitive impairment”) in Table S2.

### 2.3. Study Selection

All articles in this review were peer-reviewed between the years 2002 and 2021. The inclusion and exclusion criteria (Table 1) were determined by conducting a review meeting regarding the selection of studies. Per the guidelines for reviewing the scope of the subject, two reviewers (the first author and corresponding author) examined 83 studies, including abstracts and texts, to improve the consistency of the search. Disagreements between the two researchers regarding study selection were resolved through additional discussions and agreements. In the event of any disputes, a third reviewer was to be consulted, but this was not required.

### 2.4. Data Charting

We extracted data from the included publications using a standardized data charting form while maintaining the terminology from the papers. The form included information on the authors, year of publication, country of origin, aim, purpose, population and sample size, DST intervention, design, method, recipients or observers, methods for sharing stories, and results (Table A1, Appendix A).

### 2.5. Collating, Summarizing, and Reporting the Results

We employed an inductive approach to thematically organize and summarize the results from the included papers to answer the research questions [21].

### 2.6. Quality of Included Articles

The Mixed Methods Appraisal Tool (MMAT) was used to assess the methodological quality of the included studies [22]. The MMAT is a critical evaluation tool created for the evaluation phase of systematic mixed study reviews of empirical research, or reviews that incorporate studies using mixed, qualitative, and quantitative methodological approaches. It comprises distinct sets of criteria to assess study validity in each of the study designs. Although it is recommended to report on each criterion scored in the appropriate section of the appraisal tool in order to provide a true value of the quality of each study, for this review, an overall score was calculated from a mean score of all items in the pertinent section of the checklist. Each “Yes”, “No”, and “Unclear/can’t tell” acquired a nominal value of 2, 0, and 1, respectively. Nevertheless, the authors concurred that the scoring enables the reader to consider the relative quality of the publications. There are no cut-off scores for the MMAT when identifying the quality of research. Notably, all papers are included and handled identically for the scoping review, regardless of quality in Table S1a–c.

## 3. Results

**Figure 1:**
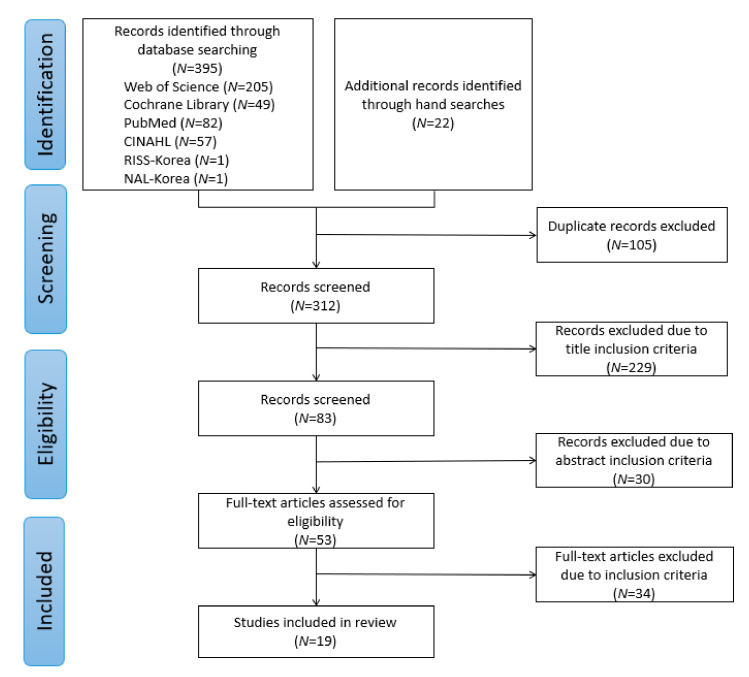
Flow diagram of study selection.

Database and manual searches yielded 395 publications. We screened the titles and abstracts of 259 publications after removing 105 duplicates. Based on the inclusion and exclusion criteria, the full text of 53 publications was read, 34 publications were excluded, and 19 were included in the review (see Figure 1).

### 3.1. Description of the Included Studies

Nineteen papers were included. The studies were conducted in Australia (*n* = 1), Brazil (*n* = 1), Canada (*n* = 5), Finland (*n* = 1), Germany (*n* = 1), Sweden (*n* = 1), Turkey (*n* = 1), the United Kingdom (*n* = 5), and the United States (*n* = 3). They employed qualitative (*n* = 12), mixed methods (*n* = 6), and quantitative (*n* = 1; randomized controlled trial) designs. Mixed methods studies mainly employed qualitative methods or open-ended questions post-intervention (*n* = 14), and quantitative studies involved questionnaires. Four, six, and nine studies were published between 2011 and 2013, 2014 and 2017, and 2018 and 2021, respectively.

Study quality was determined to be high and appropriate overall based on the MMAT’s quality assessment [22]. Two independent reviewers that contributed to the rating appraisal reached a consensus through debate.

### 3.2. Participants

The sample size of the included studies ranged from three to eighty-eight older adults; in six papers, the sample sizes were ten or fewer. The studies included patients with dementia (*n* = 5), patients with Alzheimer’s disease (*n* = 2), stroke survivors (*n* = 1), patients with an unreported diagnosis (*n* = 2), and patients with no diagnosis (*n* = 9).

The older adults engaged in DST across several age groups (50–99 years). Studies were conducted with elementary school-aged to college-aged students (*n* = 7), caregivers or trained volunteers (*n* = 3), spouses and/or families (*n* = 3), and other study facilitators. Appendix A presents the characteristics of the included studies.

### 3.3. Themes

1. How is a DST intervention structured and applied to older adults?

The results regarding application structure were framed as “connecting”, “mediating”, and “setting” in order to answer the research question on how storytelling intervention was performed using digital technology (see Table 2).

#### 3.3.1. Connecting

Individuals from various generations accompanied older adults in DST tasks. Older adults shared stories through DST with families, young people, and caregivers. After attending DST exhibitions, many community residents reported that they better understood and connected with the older adults [2,3,4,10,11,23,24,25,32,33,34].

#### 3.3.2. Mediating

The DST tool included pictures, audio, video, music applications, and GPS. The older adults and their partners engaged in DST using a tablet PC. For those unfamiliar with computers, DST was made easier through the use of a touchscreen device.

#### 3.3.3. Setting

The DST implementations adopted varied forms, such as websites, applications, or software. The stories were posted on social networking sites or made available on DVDs for participants and their families who wanted to view the stories after they were made. Workshops (*n* = 10), school processes (*n* = 3), and one-on-one sessions (*n* = 5) were used. Additionally, researchers visited seniors’ clubs and engaged in DST using various methods, such as teaching people.

### 3.4. Thematic Groupings

2. What are the effects of DST intervention on older adults?

The effects of DST intervention on older adults with or without mild cognitive impairment (MCI) were framed as follows (see Table 3).

#### 3.4.1. Promoting Older Adults’ Mental Health 

In 14 studies, older adults that experienced DST programs reported increased confidence, self-esteem, and self-efficacy, as well as reduced depressive symptoms. The older adults in these studies also reported improved mental health. The DST method was perceived as simple, effortless, and easy, thus enabling older adults to learn new things and undertake creative challenges that gave them a sense of pride and fulfillment [11,23,26,27,34]. DST allowed them to express themselves better than before, experience more pleasure, and be confident [4,10,11,12,23,24,26,34,35]. Moreover, they reported feeling livelier [4,25,29,30]. Subramaniam and Woods [24] stated that older adults reported lower depression following a DST intervention, and their caregivers and relatives corroborated this. Some older adults, for whom DST was applied with reminiscence therapy, had difficulty reviving unpleasant memories [24,26], but most reported improved self-confidence and well-being.

#### 3.4.2. Meaningful Community Connection

Thirteen studies reported that the DST intervention helped older adults feel more connected with others than before. They enjoyed sharing their stories, talking to others, and engaging in meaningful interactions [2,4,23]. They also participated in a digital life history project [2,3,10,25,30] to share their family roots and traditions with future generations. Students [2,3,4,10,23,25] and spouses [23,25,30,31,35] who accompanied older adults in these tasks, and the older adults themselves, reported a greater sense of belonging and strengthened relationships [2,3,4,10] after performing the tasks. The social associations among the participating students improved [25]. Sharing the completed stories on public platforms enabled them to understand each other better and have meaningful interactions about their experience [2,3,10,11,23,25,27,31,32,34,35]. Additionally, some participants felt that the shared experiences helped to create a social space [2,3,4,25,27,30]. Community partners, such as the local older adult housing community and historical commission, made the communities livelier than before.

#### 3.4.3. Achieving Digital Literacy

Per seven studies, older adults learned new things, became accustomed to digital technology, and gained more access to ICT. Older adults were more willing to learn new skills [3,10,12,26,35] when creating their own stories or expressing their lives by further thinking about them and re-imagining them [10,11,12,28,31,35]. This was achieved by turning still photographs into live pictures, thus adding the elements of sound and life [10,28,31]. Such activities with a creative focus can be particularly effective for people with dementia, as indicated by DST’s positive effects. During interviews before the project, older adults were worried about participating in the storytelling program due to unfamiliar and digital technology being used [11,23,26]. However, even though they operated the technology for the required time, they reported that they were happy to create stories [3,23,31]. By learning how to use computers and tablet PCs to shoot images or videos and becoming accustomed to using digital skills, older adults improved their level of digital literacy [3,23,31].

#### 3.4.4. Mitigating Negative Ageism

Five studies reported that young people’s (elementary to graduate students) perceptions of older adults changed positively, reducing their prejudices and discriminatory behaviors. In these studies, the students engaged in DST with older adults [12,23,25,32,33]. As participants, young people felt empathy toward older adults when they could relate to some of their experiences, thus forming a more positive perspective. It demonstrated that they were not simply cranky older adults, but were individuals with a wealth of knowledge and life experience who have much to impart to younger generations [33]. Older adults’ life experiences and social and work values deepened young adults’ understanding and reduced biases toward them [23].

#### 3.4.5. Enhancing Intellectual Ability

Across the six studies, DST improved memory and knowledge in older adults through the process of recalling their forgotten memories [11,24,26,27,30]. The participants recalled their past while thinking about the stories they could tell their children, nieces, or nephews [3,24,26,30]. Watching videos made by other older adults also enabled them to remember their past and revive additional memories [26]. Subramaniam and Woods [24] used the autobiographical memory interview extended version (AMI-E) and found that all participants’ knowledge scores improved. Although older adults and those with dementia had limited memory capacity, they could maintain cognitive function through repetitive learning [27]. Mental activity also helped maintain their skills and knowledge; however, the impact of dementia was evident in their memory loss and lethologica [26,27].

## 4. Discussion

This scoping review mapped and assessed published studies on DST among older adults and confirmed its application structure and effectiveness for them. This review summarizes the methods used to create digital stories, factors of digital story products, and outcomes and implementations of DST activities. The outputs were characterized as “connecting”, “mediating”, and “setting” in order to answer the research question concerning how digital technology has been structured to implement storytelling interventions. According to this structure frame, DST interventions utilizing various media and contexts might be developed by incorporating a range of participants. Although relatively simple, DST can stimulate an individual’s cognitive and sensory functions, resulting in various indirect experiences. The development of ICT has made it easy to create videos using a tablet PC or desktop application. DST can be used repeatedly, regardless of whether the older adults are community or nursing home residents. Furthermore, creating an online platform where older adults can share stories may encourage interactions with their family and community. Sharing life stories can create opportunities that allow members of the community to understand the experiences of others [3].

Engaging in DST was able to provide great vitality and prevent older adults living in nursing homes from feeling as though they were “dead weight” [4]. Moreover, they could experience improved connectedness and well-being through non-contact DST during the COVID-19 pandemic. Reciprocal story sharing can reduce feelings of isolation through the forming of social connections and new relations [36]. Although face-to-face interactions were reduced due to COVID-19, the non-contact method of using the platform could create a new way of communicating for them.

In this review, we found that the most common effects of DST on older adults included the promotion of mental health, an increased amount of meaningful community connections, greater digital literacy, the mitigation of negative ageism, and enhanced intellectual ability. Since the effects of DST intervention were identified at the individual and community levels, it is possible to further develop DST interventions and examine their effects across a range of outcomes.

The older adults and young people improved their understanding of one another through DST, thereby reducing ageism and improving young people’s perceptions of older adults [27,28,35]. Thus, DST can help improve relations between older and younger generations through meaningful interactions [33,35]. We further recommend randomized controlled trials using intergenerational DST interventions and systematic reviews to examine the community-level outcomes of DST interventions.

There were twelve qualitative studies and six mixed methods studies. Qualitative evaluations were conducted using semi-structured interviews and open-ended questions. Mixed methods studies quantitatively evaluated older adults with MCI and dementia, as well as their spouses, family members, and caregivers. DST was found to be helpful in managing older adults’ depression and quality of life [4,29]. The self-assessment results of the participants with cognitive impairment may be unreliable as patients may have difficulty in quantitative evaluation because their short-term memory is relatively poor. However, the increased frequency of participation in the intervention program, expressions of smiling faces, or increased food intake can be evaluated. The studies conducted a before-and-after evaluation for those caregivers or researchers who observed the participants. Alternatively, an objective effect may be derived if the intervention is filmed or evaluated [24,31].

There is no established framework for DST programs. It is up to the creator to depict the story they wish. Some programs were created by applying recall therapy, though participants could also talk about their daily lives and convey their thoughts. The participants reported similar outcomes even though they had different baseline levels of cognitive impairment. They discussed the following: recovery of cognitive ability, reduction of depressive symptoms, improved mental health, and harmony among generations. The “old adult” creates a story, but various outcomes may be achieved depending on the generation involved. The study design may be based on this principle. The existence of diversity limits the ability to conduct rigorous quantitative research within such a framework. By revealing the core concept and its theoretical and practical implications, factors predicated upon the results of various studies, developing a program based on this framework is necessary. 

### Limitations

There may be many articles where older adults use digital media to tell stories. However, several studies may have been excluded because of the limited scope of the literature search. This research is limited to English and Korean language publications. There may be other DST papers written in other languages that were not included in this review. 

## 5. Conclusions

This scoping review can contribute to the use of DST in improving the quality of life and mental health of older adults with or without cognitive impairment. DST can be used to easily create a digital story anywhere with tools that users prefer and songs, photos, and paintings that they like. It can be applied to older adults using easy-to-use devices, such as tablet PCs and laptops, without any significant preparation and has the advantage of generating DST even in non-face-to-face situations. The older adults who experienced DST intervention gained confidence in digital technology, increased their e-literacy, decreased negative emotions such as depression and loneliness, and experienced intergenerational interaction. It is anticipated that extending DST intervention to older adults will improve their quality of life, increase their energy, and support their mental health. Furthermore, if the story created is shared with the community and the younger generation, it will reduce the gap between generations and help them to understand each other better. It is necessary to develop intervention programs tailored to the varied needs of older adults with different health conditions; furthermore, interventions to enhance community integration must be developed soon. Family members or young people are encouraged to appropriately participate in DST interventions for older adults to facilitate access to digital technology.

## Figures and Tables

**Table 1:** Inclusion and exclusion criteria.

Criterion	Inclusion	Exclusion
Type of studies	Qualitative, quantitative, and mixed methods studies published in peer-reviewed journals	Letters, comments, abstracts, editorials, doctoral theses, or any type of review
Period	From 1 January 2002, until 19 December 2021	
Language	English, Korean	
Type of participants	Older adults aged 50 years or older with mild cognitive impairment (MCI) or dementia	Cancer, patients with other diagnoses
Phenomenon of interest	Studies that include narrative-type media production that delivers stories using video, photo, audio, sound media, etc.	Narrative research without the use of digital media

**Table 2:** Application structure for digital storytelling intervention.

Dimension	Components	Study	Number of Articles
Connecting with the older person’s companion	Older person	[3,4,10,11,23,24,25,26,27,28,29]	11
	Family	[2,3,10,11,23,25,27,29,30,31]	10
	Student (young person)	[2,3,4,10,12,23,25,32,33]	9
	Community member	[2,3,10,25,26,31,32,33,34]	9
	Caregiver	[11,24]	2
	Trained volunteer	[22,28]	2
Mediating DST tool	Picture	[2,3,10,11,12,23,24,26,27,28,29,30,31,32,33,34,35]	17
	Audio	[2,3,10,11,23,24,25,26,27,28,29,32,33,34]	14
	Video	[2,3,10,23,24,25,26,27,28,29,30,33,34]	13
	Music	[2,3,4,10,24,25,26,29,30,32,35]	11
	Application	[2,4,12,26,29,31,32]	7
	Touchscreen device	[26,31,35]	3
	GPS	[30]	1
Setting DST implementation	Software	[2,3,4,10,11,24,26,29,30,33]	10
	Workshop	[2,3,10,11,12,25,27,32,34]	10
	Website	[2,3,10,26,31,33,34,35]	8
	One-on-one session	[2,3,10,23,25,26,31,35]	8
	School	[12,23,25]	3
	Social network	[23,30]	2

**Table 3:** Articles included in thematic groupings.

Theme	Study	Number of Articles
Promoting older adults’ mental health	[2,4,10,11,12,23,24,25,26,27,29,30,34,35]	14
Meaningful community connection	[2,3,4,10,11,23,25,27,30,31,32,34,35]	13
Achieving digital literacy	[3,10,11,12,28,31,35]	7
Mitigating negative ageism	[3,23,25,32,33]	5
Enhancing intellectual ability	[3,11,24,26,27,30]	6

## Data Availability

Not applicable.

## Supplementary Materials

The following supporting information can be downloaded at: https://www.mdpi.com/article/10.3390/ijerph20021344/s1, Table S1a: Quality assessment of the qualitative studies included in the review; Table S1b: Quality assessment of the quantitative randomized controlled trials included in the review; Table S1c. Quality assessment of the mixed-methods studies included in the review; Table S2: Search String per Database.

## Appendix A

**Table A1:** Characteristics of the included studies.

Author, Year, Country	Aim	Sample	Digital Storytelling Application, Delivered Mode	Design and Methods	Results
Bentley, Basapur, Chowdhury [30] 2011 USA	To promote family bonds by fostering communication between generations and to improve users’ relationships with daily places in their lives using asynchronous communication.	Ten older adults (aged 50+); the older relatives of adults in their 20s to 40s. Five were from South Florida and five from Chicago, though not living with the main participant. Ten young participants from Chicago.	The younger participants either downloaded the app onto their current Android phone or switched their SIM card to a new Android phone. They called the voicemail system every time they had a story to report on for four weeks. Initially, older adults recorded stories utilizing the study’s web interface. They were instructed to create a minimum of five videos for their younger relatives utilizing our system’s website. They chose the topic, setting, and storytelling style.	Qualitative. Observational method. Interviews with younger relatives regarding their story sharing and communication methods.	The mobile phone is an amazing medium for encouraging reminiscences and developing a stronger sense of family unity. The outcomes of storytelling include the elicitation of powerful emotions and memories, increased communication, the expansion of storytelling to family and friends, re-engagement with the city through family history, and strengthened family relationships through storytelling.
Brandão Bauer, Haas et al. [4] 2021 Brazil	During the COVID-19 pandemic in the south of Brazil, the aim was to examine the viability of an intergenerational remote intervention program meant to increase the well-being and social connection of vulnerable older adults, particularly people with aphasia and dementia.	Thirty-four participants (stroke survivors with expressive aphasia); fourteen females. A total of 83% and 7% of participants had high and medium attendance to the program, respectively.	Through sessions involving clowning, storytelling, dancing, and cooking-related activities, the Playful Living program promoted the health and social connectedness of vulnerable older persons. Each participant was a member of a WhatsApp group containing other participants and teams. Everyone had to schedule at least one WhatsApp video call weekly. The livestream sessions engaged a consistent cross-generational peer group.	Feasibility study. Mixed methods approach. Open-ended questions. Questionnaire and workshop format.	This pilot study illustrated the feasibility and acceptability of providing remote activities for older persons, especially those with cognitive impairments. The initiative demonstrated that virtual environments might be used compassionately and that interdisciplinary teams can build meaningful connections in this new context. This study did not involve cognitive evaluations of participants nor a comparison of before and post-intervention measures. A significant number of participants and family members did not complete the questionnaires and assessments, citing family health and stress-related reasons related to the pandemic.
Critten, Kucirkova [26] 2017 UK	To examine and theorize the role of digital multimedia personalization in stimulating and sharing the long-term memories of individuals with mild to moderate dementia.	Three older adults (aged 63 to 94) with mild to moderate dementia, accompanied by their husbands.	The Story app facilitated selecting and arranging images based on the participant’s description of who or what was significant in each image (typed as a caption or audio-recorded). Printed version of the completed story was a trigger for later reminiscence and discussion with family.	Qualitative methods. Case study. Semi-structured interviews.	The study found that using story-making software was able to facilitate the elicitation, preservation, and sharing of special memories for three individuals with varying stages of dementia. The approach was enjoyable for all three participants (and their caretakers) and resulted in improved self-confidence, autonomy, and self-respect.
Elfrink, Ullrich, Kunz, et al. [29] 2021 Germany	To examine the efficacy of the Online Life Story Book intervention on neuropsychological symptoms in individuals with mild dementia, as well as the distress and quality of life of their primary informal caregivers.	Online life story group: 23 participants. Of these, four failed to complete the intervention. A group of 19 served as a control. Of these, two failed to complete the intervention.	Approximately five meetings conducted over the course of eight to ten weeks. The Online Life Story Book (OLSB) is a web-based program for health care. After placing memories such as life events, anecdotes, photographs, films, voice fragments, music, recipes, preferences, and activities on the timeline, online books can be produced. QR codes enabled internet access to multimodal memories. Volunteers assisted individuals with dementia and their caregivers in completing the OLSB.	RCT (s randomized controlled experiment with two arms and three measures).	There were no significant changes between the experimental and waitlist control conditions, except for the self-rated suffering of informal caregivers.
Freeman, Martin, Nash, et al. [2] 2020 Canada	To provide an opportunity for meaningful contribution to their community by conserving their cultural identity and fostering intergenerational interactions between older and younger adults.	Thirteen older adults (aged 50+) and thirty-one primary school seniors.	Four weeks, ten 2.5-hour-long sessions utilizing the video editing software WeVideo (an internet video editing application that can be used from anywhere). Three workshop sessions were devoted to introducing the participants to the concept of digital storytelling, getting to know one another, and exchanging stories in large and small groups. Students began storyboarding and utilizing WeVideo technology in the fourth session, which was explicitly geared toward them. Any components of this research were governed by the Ownership, Control, Access, and Possession (OCAP^®^) principles (First Nations Information Governance Centre, 2014). As a result, all digital videos recorded remain the property of the older adults and students of the Nak’azdli community.	Qualitative method. Workshop format. Semi-structured interviews. Focus group interviews.	Not only were connections created between the younger participants and older adults, but also between the older adults themselves, and their profound role as teachers and keepers of community knowledge was acknowledged. Community members noticed that this public acknowledgment and the weekly involvement with students positively affected the older adults’ mental health and well-being. Developing and fostering intergenerational ties through a digital storytelling workshop in which older adults and youth share cultural and experiential stories is essential for maintaining older adults’ wisdom and knowledge for future generations.
Hausknecht, Vanchu-Orosco, Kaufman. [10] 2017 Canada	To continue the 2016 Computer Supported Education conference conversation regarding creating and evaluating a series of digital storytelling workshops for older individuals.	Forty older adults (aged 55+). There were three cycles of the program, each with seven groups of four to ten participants.	The program was structured according to two distinct phases: story creation and digital creation (ten weeks total). The software was straightforward to use, simple to access from numerous places, had publishing choices, and was suitable for the demographic and workshop setting. Participants received a more comprehensive understanding of the process as they were introduced to the digital storytelling software earlier and allowed to spend additional time with facilitators and tutorials outside the session. They were also given small lectures on the software’s various features (such as adding photographs and videos to the “main track” and modifying narration in the “audio track”) and were encouraged to experiment with it.	Mixed methods approach. Questionnaire and workshop format. Open-ended questions. A five-point scale asking participants what they liked most and what could be improved about the workshop.	Participants enhanced their digital literacy and proficiency. Positive aspects included sharing and connections with others, creating/learning of digital stories, and facilitation (achieved via shared social experience, personal expression by learning story creation, and helpful facilitation). Time, or the lack thereof, appeared to be the most significant issue that required addressing.
Hausknecht, Vanchu-Orosco, Kaufman. [3] 2018 Canada	To investigate the potential benefits and experiences of older persons who took a digital storytelling course, as well as to acquire a deeper knowledge of the socioemotional components of the process.	Course: 88 participants (older adults); 68 females (81.9%), aged 55–99 years. Immigrants: 43 (51.2%). Attendance: 47 community members (family, friends, and participants) attended one of the “Sharing Our Stories” events.	The program duration was ten weeks (some older courses lasted eight or nine weeks). Participants met weekly for two-hour sessions in addition to additional tutorial sessions. Course enrolments fluctuated in size. During the technical portion of the digital story development process, at least one facilitator was present for every two to three participants.	Mixed methods approach. Focus group interviews (four groups). Semi-structured interviews.	The results of the focus group interviews revealed a plethora of reported social and emotional benefits associated with the course’s digital story creation process. Three key themes emerged: social connection through shared experience and narrative, recollection and life reflection, and the creation of an enduring legacy. Attendees of a “Sharing Our Stories” event said that the stories were meaningful, well-written, and elicited various emotions. Digital storytelling may help digital storytellers strengthen their connections with others and themselves. The process of studying the past to produce a digital story that might serve as a legacy that future generations can connect to may also extend this connectedness over time.
Hewson, Danbrook, Sieppert [33] 2015 Canada	To investigate (a) student experiences with digital storytelling and its perceived value as a tool for social work and (b) student thoughts on intergenerational application with older persons.	Seven older adult (in their late 50s to early 70s) storytellers; eight social work students.	Three-day workshop involving multiple generations. Older adults created individual stories based on the theme “stories of home.” Students: studied Digital Storytelling with older participants and assisted them.	Mixed methods approach. Semi-structured interviews. Questionnaire.	Students cited the intergenerational component as a highlight of the course, which enhanced their understanding of older adult challenges and their ability to work with aging populations. Older adults appreciated working with the students, which enhanced their understanding of the younger generation.
Jaakola, Ekström, Guilland [12] 2015 Finland	To encourage changes in older adults’ attitudes and behaviors regarding mobile devices and app usage.	Five interviews and the creation of personalities were conducted with the following participants: 17 persons, 14 females, with a mean age of 63.5 (range: 59–73) years. Co-designing and piloting a training program with fifty older adults aged 61–85 years. The range of participation in each session was between 11 and 19.	Co-designing and piloting a 12- to 15-hour training course; each session lasted three to four hours. Three straight weeks of once-per-week meetings were held.	Multi-method. One-on-one semi-structured interviews.	Training can assist older adults in integrating new technologies in novel and frequently surprising ways. However, fresh and creative techniques are required to encourage older adults to learn to use and fully exploit the vast array of tablet PC capabilities. Establishing a training idea was essential for an iterative and collaborative approach.
Karlsson, Axelsson, Zingmark, Fahlander, et al. [31] 2014 Sweden	To investigate the process of acceptance and integration of a digital image diary as a tool for a person’s recollection and discussion of daily life events.	Twenty patients with Alzheimer’s disease (age range: 72–81 years old; Mini Mental Status Examination scores: 17–25) and their family members.	Digital graph journal created as part of the MemoryLane project using a SenseCam, a modified smartphone, and a home station. Using the camera and the smartphone to document daily occurrences and activities. The frequency of camera use was up to the discretion of each pair. The family member connected the camera and smartphone to the computer to upload the captured images in the evening. When reviewing the images, it was possible to include both a header and descriptive text, and a date was appended automatically. The family member was advised to ask the person with dementia to recount the associated incident when reviewing the images.	Explorative. Multiple case studies. Individual semi-structured interviews. Survey format.	No correlation was found between cognitive function and usage. The personal desire to begin using the digital photograph diary (DPD) was tied to an anticipated utility. The capacity to learn and comprehend the operation of the DPD was facilitated by actual user experience. Acceptance and integration were a process with no beginning and end. Personal expectations, the capacity to learn and comprehend the instrument, actual use, assistance, and perceived utility were all significant elements in this procedure.
Loe [25] 2013 USA	Describe the implementation of the Digital Life History Project as an example of intergenerational community-based learning.	Students are “matched” with local community members randomly (aged 55+).	The study found that 4 to 10 weeks were required to make the final product. The major purpose of the Digital Life History Project was to create a three- to five-minute graphic biography of a person’s life. Students and learning partners collaborated throughout the semester to create, direct, produce, and edit their digital stories. This relationship could result in a vast array of individual, societal, and community effects. The objective of the initial interview was to become acquainted and develop trust and rapport. During the remaining weeks of the semester, the learning partners edited, finalized, and recorded the audio narrative in collaboration (in most cases, the older adult partner chose to narrate their life story). Finalizing the digital story could involve adding animation, sound effects, transitions, titles, and credits and synchronizing the audio and video. Members of the university and the greater community were invited to attend the screening of the digital tale. Each participant took home a DVD to share with family and friends.	Qualitative workshop (in classroom learning). Background in empirical research and methodological training. Surveys. Interviews with semi-structured questions. Notations and observations.	Empathy, interpersonal communication, long-term planning, and attentiveness were crucial to the success of this project. Five frequent areas of growth were identified by community-dwelling older adults and student participants: establishing meaningful relationships, connecting biography and history, learning to combat ageism, plotting the next chapter, and community adoption. Notably, for many, these impacts persisted beyond the project’s semester of implementation.
McGovern [23] 2019 USA	To suggest that innovative pedagogical techniques that expand fundamental social work concepts can enhance outcomes for varied older individuals by fostering multicultural gerontology practice competency.	Twenty-five undergraduate students (age range: 20–57 years old) and twenty-five multicultural participants above the age of 65.	One-semester, one-class gerontology practice course with five 1.5-hour portions of the fourteen 2-hour and 40-minute weekly sessions.	Evaluation of a project. Case study. Semi-structured interviews. Students’ anonymous written responses.	Three strengths emerged from student participation in a digital storytelling experiential learning project, as revealed by their reflections: a deeper comprehension of late life and social work ideals, an enhanced interest in gerontology practice, and a greater commitment to learning. A greater appreciation for social connection also evolved among students and across generations.
Schoales, Jones-Bonofiglio, Stroink, et al. [32] 2020 Canada	Through creating a digital story, investigate older persons’ unique perspectives on aging successfully in their community and at home.	Ten older adults within the community (58–99 years old). Eight people (interview). Six people (focus group interview). Five were female.	Three-day event with the theme of well-being and a 35-minute video. The digital storytelling session was facilitated by GIANTs project professionals and volunteers. Each participant was invited to write a personal narrative demonstrating their capacity to age gracefully in the community and was aided in developing a video illustrating this. Volunteers and facilitators assisted participants with audio recording. Facilitators revised each finished digital story with participant participation. Following the workshop, everyone could view the footage collectively.	Qualitative method. Workshop format. Semi-structured interviews. Individual and focus group interviews.	Participants highlighted parallel subthemes of efficacy, engagement, enjoyment, and interpersonal interactions while discussing their definitions of well-being and experiences with digital storytelling. Utilized Seligman’s PERMA model of well-being, which includes: P/Positive Affect, E/Engagement, R/Relationships, M/Meaning, A/Achievement.
Simsek, Erdener [28] 2012 Turkey	To explore whether basic digital visual technologies can be developed within the scope of digital storytelling workshops.	Three older adults (60–70 years old), females.	Three-day event. Using reminiscence therapy, older individuals explored a park, took photographs, and related stories. At the second meeting, a media expert introduced storyboarding. They then created pictures by mixing videos with speech recordings.	Qualitative methods. Pilot study. Workshop format.	As the three individuals were already acquainted, no appropriate evaluation was conducted. Experimenting with DST as a mobile workshop practice to increase the digital inclusion of older females, with an emphasis on the digital visual skills education component as a study area, was crucial for observing the mechanisms at play in co-learning processes among older individuals.
Sljivic, Sutherland, Stannard, et al. [34] 2021 Australia	To determine whether the development of empathy through digital storytelling influences the attitudes of young people toward older adults. An exhibition of digital storytelling expressing the experiences of older adults.	Twelve older adults (aged 65+), including six females.	Older adults generated digital stories, but no specific techniques were validated. Using the exhibition featuring their stories, the evolution of young people’s attitudes toward older adults was analyzed.	Mixed method. Pilot study. Semi-structured interviews. Questionnaire.	According to the pre–post Refined Aging Semantic Differential [RASD] evaluation of young people, perceptional differences regarding older adults have improved. The effects of confronting older individuals’ preconceptions include a shift in attitude, the formation of empathy through shared experiences with the storyteller, and the confirmation of the digital story’s validity.
Stenhouse, Tait, Sumner [11] 2013 UK	As part of an educational package for student nurses, practice digital storytelling with dementia patients in the early stages.	Eight older adults with early-stage dementia; one caregiver.	Four-day workshop. Day 1: introductions and the display of sample Patient Voices digital stories. Day 2: discussion of the workshop process, writing the script, and feeding all images into the computers. Day 3: discussion of the workshop process, writing the script, and feeding all images into the computers. Listen to voiceovers while utilizing video editing software to construct a digital tale. Day 4: Participants, staff, and facilitators assemble to present all stories. The group was requested to discuss their experiences.	Qualitative method. Observation during the workshop. The four facilitators gathered at the end of each day to discuss the day’s events. The reflecting sessions’ content was grouped thematically.	This workshop proved that dementia patients in the early stages could engage in creative activities integrating technology and narrative. As they participated and developed digital stories, participants confronted numerous challenges. A relationship based on the individual assisted them in overcoming these obstacles. Participants observed positive improvements, including increased confidence, enhanced communication, a sense of purpose, and a strengthened sense of connection.
Subramaniam, Woods [24] 2016 UK	To examine the efficacy of dementia patients utilizing multimedia digital life storybooks in nursing homes from the perspectives of dementia patients, their family members, and care staff.	Six older adults with dementia (73–89 years old) living in care homes, one with mild dementia and two with moderate dementia.	Seven to ten weeks (mean 8.3), four females. Single-case methodology, researcher functioning as co-editor (research team). Some older adults who developed life storybooks generated digital versions. Several older adults who developed life storybooks also created digital life storybooks, which featured records in chronological order, career, images initially included in the book, and visual or musical resources that the older adults enjoyed. During the creative process, participants and family members confirmed and requested modifications. Patients and family members gave the narrative. It was converted to DVD format and distributed to the participants, their families, and care home employees.	Mixed method. Multiple case studies. Participatory design. Semi-structured interview. Questionnaire format.	Tests were conducted for participants, caregivers, and family members (quality of life, autobiographical memory, and depression scores improved). Five participants had improved quality of life and autobiographical memory. All individuals’ depression levels improved or remained stable. According to a thematic analysis, participants, families, and staff considered digital life storybooks a very valuable tool for evoking fond memories and happy feelings. The lack of a control group and blinded post-intervention assessments are limitations of the study.
Sweeney, Wolverson, Clarke [35] 2021 UK	To study the differences and shared experiences of people with dementia and their spouses through the creation of a digital biography.	Ten couples (a person with dementia and their spouse), 74–91 years old.	Making a digital storybook and conducting interviews for six weeks. Using images, films, and music, a program was designed for older adults with dementia. It is compatible with laptops, tablet PCs, and smartphones.	Qualitative. Constructivist and grounded theory. Epistemological position. Semi-structured interviews.	“Creating a life storybook is a monumental task”, “Looking back and looking forward: The emotional journey”, “Whose story is it and to whom does it belong?”, and “Challenges of utilizing technology to create the life storybook” were analyzed thematically. The experience benefited those with dementia and their spouses. It highlighted the significance of couple hood in constructing a life story. Life story work is not simple; experience is not always positive and working on life stories can lead to despair and a sense of loss, contrary to the researcher’s hypothesis.
Ward, Thoft, Lomax, et al. [27] 2020 UK	Utilizing the creativity involved in photography and storytelling to gain an understanding of students’ experiences with dementia via the development of diverse methodologies.	Twelve older adults (67–83 years old), seven with Alzheimer’s. Five were female.	Conducted for four weeks. Proceedings occurred at the Dementia School for the Elderly. Using a direct camera, participants took photographs of places and objects that the older adults enjoyed around the school or their homes and witnessed them narrate stories using the photographs. Stimulating and explaining new memories and sharing them with family.	Qualitative method. Workshop format. Photo-elicitation and storytelling methods. Open-ended questions.	Themes: school activities, knowledge and skills, the value of friendship, and what it means to live with dementia. The questions were designed to emphasize emotional and personal experiences rather than recall.
