# Supplementary material for: Digital Storytelling as an Intervention for Older Adults: A Scoping Review
Source: Int J Environ Res Public Health. 2023 Jan 11;20(2):1344. doi: 10.3390/ijerph20021344 (PMC9859096; doi:10.3390/ijerph20021344)
Supplement: Supplementary file 1 [file ijerph-20-01344-s001.zip › ijerph-2063459-supplementary.pdf]

**Table S1a.** Quality assessment of the qualitative studies included in the review.					
	**Questions from the Mixed Method Appraisal Tool (MMAT), version 2018**				
**Included studies**	Is the qualitative approach appropriate to answer the research question?	Are the qualitative data collection methods adequate to address the research question?	Are the findings adequately derived from the data?	Is the interpretation of results sufficiently substantiated by data?	Is there coherence between qualitative data sources, collection, analysis and interpretation?
Bentley, et al. (2011)	Yes	Yes	No	Yes	No
Critten, Kucirkova(2017)	Yes	Yes	Yes	Yes	Yes
Freeman (2020)	Yes	Yes	Yes	Yes	Yes
Jaakola (2015)	Yes	Yes	No	Yes	No
Karlsson(2014)	Yes	Yes	Yes	Yes	Yes
Loe (2013)	Yes	Yes	Yes	No	Yes
McGovern (2018)	Yes	Yes	Yes	No	No
Schoales (2020)	Yes	Yes	Yes	Yes	Yes
Simsek.(2012)	Yes	Can't tell	Can't tell	Can't tell	Can't tell
Stenhouse. (2012)	Yes	Yes	Yes	Yes	Yes
Sweeney (2020)	Yes	Yes	Yes	Yes	Yes
Ward (2020)	Yes	Yes	Yes	Yes	Yes

**Table S1b.** Quality assessment of the quantitative randomized controlled trials included in the review.					
	**Questions from the Mixed Method Appraisal Tool (MMAT), version 2018**				
**Included studies**	Is randomization appropriately performed?	Are the groups comparable at baseline?	Are there complete outcome data?	Are outcome assessors blinded to the intervention provided?	Did the participants adhere to the assigned intervention?
Elfrink, (2021)	Yes	Yes	Yes	Yes	Yes

**Table S1c.** Quality assessment of the mixed-methods studies included in the review.					
	**Questions from the Mixed Method Appraisal Tool (MMAT), version 2018, for the qualitative part**				
**Included studies**	Is the qualitative approach appropriate to answer the research question?	Are the qualitative data collection methods adequate to address the research question?	Are the findings adequately derived from the data?	Is the interpretation of results sufficiently substantiated by data?	Is there coherence between qualitative data sources, collection, analysis and interpretation?
Brandao. (2021)	Yes	Yes	Yes	Yes	Yes
Hausknecht (2017)	Yes	Yes	Yes	Yes	Yes
Hausknecht (2018)	Yes	Yes	Yes	Yes	Yes
Hewson (2015)	Yes	Yes	Yes	Yes	Yes
Sljivic. (2021)	Yes	Yes	Yes	Yes	Yes
Subramaniam. (2016)	Yes	Yes	Yes	Yes	Yes
	**Questions from the Mixed Method Appraisal Tool (MMAT), version 2018, for the quantitative part**				

	Is the sampling strategy relevant to address the research question?	Is the sample representative of the target population?	Are the measurements appropriate?	Is the risk of nonresponse bias low?	Is the statistical analysis appropriate to answer the research question?
Brandao. (2021)	Yes	Yes	Yes	Yes	Yes
Hausknecht (2017)	Yes	Can't tell	Yes	Yes	Yes
Hausknecht (2018)	Yes	Can't tell	Yes	Yes	Yes
Hewson (2015)	No	Can't tell	Yes	Yes	Yes
Sljivic. (2021)	Yes	Yes	Yes	Yes	Yes
Subramaniam. (2016)	Yes	Yes	Yes	Yes	Yes
	**Questions from the Mixed Method Appraisal Tool (MMAT), version 2018, regarding mixed method design**				
	Is there an adequate rationale for using a mixed methods design to address the research question?	Are the different components of the study effectively integrated to answer the research question?	Are the outputs of the integration of qualitative and quantitative components adequately interpreted?	Are divergences and inconsistencies between quantitative and qualitative results adequately addressed?	Do the different components of the study adhere to the quality criteria of each tradition of the methods involved?
Brandao. (2021)	Yes	Yes	Yes	Yes	Yes
Hausknecht (2017)	Yes	Yes	Yes	Can't tell	Yes
Hausknecht (2018)	Yes	Yes	Yes	Can't tell	Yes
Hewson (2015)	Yes	Yes	Yes	Can't tell	No
Sljivic. (2021)	Yes	Yes	Yes	Yes	Yes
Subramaniam. (2016)	Yes	Yes	Yes	Yes	Yes

Table S2. Search String per Database (2002.1.1~2021.12.17).

Database	*Number of result*	*Search String*
Pubmed	*82*	(story* [Title/Abstract]) AND (digital* [Title/Abstract] OR ICT [Title/Abstract] OR "artificial intelligence" [Title/Abstract] OR online [Title/Abstract] OR media[Title/Abstract] OR mobile[Title/Abstract] OR platform[Title/Abstract]) AND (elder* [Title/Abstract] OR older* [Title/Abstract] OR dementia [MeSH Terms] OR dementia [Title/Abstract] OR Alzheimer [Title/Abstract] OR Alzheimer [MeSH Terms] OR "cognitive impairment" [Title/Abstract]) NOT child* [Title/Abstract]
Web of Science	205	(((TS=((dementia OR alzheimer OR "cognitive impairment" OR elder* OR older*) AND (story*) AND (digital* OR ICT OR "artificial intelligence" OR online OR media OR mobile OR platform) NOT child*)) OR TI=((dementia OR alzheimer OR "cognitive impairment" OR elder* OR older*) AND (story*) AND (digital* OR ICT OR "artificial intelligence" OR online OR media OR mobile OR platform) NOT child*)) OR AB=((dementia OR alzheimer OR "cognitive impairment" OR elder* OR older*) AND (story*) AND (digital* OR ICT or "artificial intelligence" OR online OR media OR mobile OR platform) NOT child*)) AND LA=(English)
EBSCOhost-CINAHL	57	(TI "story*" OR AB "story*") AND ((TI "dementia" OR AB "dementia") OR (TI "Alzheimer" OR AB "Alzheimer") OR (TI "cognitive impairment" OR AB "cognitive impairment") OR (TI "elder*" OR AB "elder*") OR (TI "older*" OR AB "older*")) AND ((TI " digital*" OR AB " digital*") OR (TI" ICT" OR AB" ICT") OR (TI " artificial intelligence" OR AB " artificial intelligence")" OR (TI " online" OR AB " online") OR (TI " media" OR AB " media") OR (TI " mobile" OR AB " mobile") OR (TI " platform" OR AB " platform")) NOT (TI "child*" OR AB" child*")

Cochrane	49	(story*):ti,ab,kw AND ((digital*):ti,ab,kw OR (ICT):ti,ab,kw OR (artificial intelligence):ti,ab,kw OR (online):ti,ab,kw OR (media):ti,ab,kw OR (mobile):ti,ab,kw OR (platform):ti,ab,kw)) AND ((elder*):ti,ab,kw OR (older*):ti,ab,kw OR (dementia):ti,ab,kw OR (dementia):ti,ab,kw OR (Alzheimer):ti,ab,kw OR (Alzheimer):ti,ab,kw OR (cognitive impairment):ti,ab,kw) NOT (child*):ti,ab,kw
